# 

**Published:** 1889-05

**Authors:** 


VOL. XIX. MAY, 1889. NO. 5

### Southern Medical Record.

EDITORS:

T. S. POWELL. M. D. R. C. WORD, M. D.

COLLABORATORS:

J. McF. Gaston, M. D.,
Julian J. Chisolm, M. D.,
W. P. Nicolson, M. D.,
E. Van Goidtsnoven, M. D.,
P. R. Cortelyou, M. D.,
Lindsay Johnson, M. D.,
G. G. Roy. M. D.,
A. G. Hobbs, M. D.,
W. S. Elkin, M. D.,
W. A. Crow. M. D.,
Jno. Thad Johnson, M. D.,
K. P. Moore, M. D.

**Address R. C. Word, M. D. Managing Editor, Atlanta, Ga.**

Published and entered as Second Class Matter at Atlanta, Ga.

